# Feasibility and Enjoyment of Exercise Video Games in Older Adults

**DOI:** 10.3389/fpubh.2021.751289

**Published:** 2021-11-05

**Authors:** Sara A. Freed, Briana N. Sprague, Abigail T. Stephan, Cassidy E. Doyle, Junyan Tian, Christine B. Phillips, Lesley A. Ross

**Affiliations:** ^1^Department of Human Development and Family Studies, The Pennsylvania State University, University Park, State College, PA, United States; ^2^Department of Epidemiology, University of Pittsburgh, Pittsburgh, PA, United States; ^3^Department of Education and Human Development, Institute for Engaged Aging, Clemson University, Clemson, SC, United States; ^4^Department of Psychology, Institute for Engaged Aging, Clemson University, Clemson, SC, United States

**Keywords:** aging, social isolation, social connectedness, exergame, feasibility, video games

## Abstract

**Introduction:** Several interventions have been developed to enhance social connectedness among older adults. However, little research has demonstrated their performance in a social distancing environment. Exergames are not only beneficial to older adults' physical and cognitive health, but they also allow players to interact with each other at a distance, which can reduce loneliness and increase social connection. The aim of this pilot study was to investigate older adults' perceptions of two commercially available exergames.

**Methods:** Twenty healthy community-dwelling older adults (M age = 73.30, SD = 5.95, range = 65–84 years, 80% women) were recruited in this pilot study between July 2019 and February 2020. They were asked to play two exergames for 10 min each on the Xbox One with Kinect console: Just Dance and Kinect Sports Rivals. After gameplay, they provided both quantitative and qualitative feedback on these games.

**Results:** Participants reported an average rating for exergame enjoyment. Greater enjoyment was significantly related with younger age and greater extraversion but not gender. Participants were highly motivated to do well on the games but reported lower scores for likelihood of playing these games in the future. Greater likelihood of future play was associated with younger age but not gender or extraversion. “Not aerobic or strengthen enough; not enough exertion,” and “slower movements, repetition, clear purpose of doing the exercise” were some factors that would influence their decision to buy and play these games.

**Discussion:** The preliminary results of this pilot study suggest that exergames may help address social isolation and loneliness—particularly during times of social distancing. Before applying exergames as a social isolation or loneliness intervention for older adults, study replication in larger representative studies and future work that examines important design issues related to older adults' experiences with these games is needed.

## Introduction

Social connectedness, or the “subjective evaluation of the extent to which one has meaningful, close, and constructive relationships with others” [(1) p. 43], is an essential component of well-being for older adults (2, 3). Older adults are at greater risk of feeling socially disconnected compared to younger age groups due to several age-normative isolation-inducing transitions including retirement and the subsequent loss of a social work environment, the death of close friends and family, and limited mobility as a result of age-related physical and cognitive impairments (3–5). The COVID-19 pandemic, and the resulting need for social distancing, is a non-normative transition that placed older adults at great risk for social isolation and loneliness (6–11). There is a need for activities that address social isolation and loneliness among older adults while adhering to social distancing requirements. The current study presents older adults' perceptions of two commercially available exercise video games (exergames) which hold promise for promoting social connectedness among older adults in a virtual environment.

Social connectedness safeguards against one of the most pervasive risks to older adults' health and well-being: loneliness. In fact, some scholars propose that social connectedness can be conceptualized as a lack of loneliness (1). Because loneliness has been linked to reduced life satisfaction, depression, and poor health outcomes (12, 13), enhancing social connectedness has the potential to bolster overall emotional, psychological, and physical well-being (4, 14–16). Characterized by caring/feeling cared for by others and having a sense of belonging (1), feeling socially connected is largely dependent on one's mindset; this provides space for a variety of interventions to target this construct.

For community dwelling older adults, several interventions aimed at increasing in-person connections have been developed to enhance social connectedness. The majority of these interventions have a specific aim of increasing the frequency of social contact (17, 18). In a review of 39 interventions targeting social connectedness/loneliness in older adults, O'Rourke et al. (19) found the two most prevalent intervention types focused on enhancing personal contact and implementing activity and discussion groups. Inconsistent use of measures and evaluative tools make it difficult to assess the efficacy of current interventions (19), though most studies suggest a variety of social supports, especially those that connect individuals around shared activities, can promote social connectedness and overall well-being for older adults (2, 20).

One challenge of the COVID-19 pandemic is how to promote social connectedness and well-being without the use of traditional in-person intervention formats. Exergaming has the potential to increase social connectedness, physical activity, and leisure opportunities among older adults during times of social isolation including the COVID-19 pandemic. Exergames are a type of game, often technology-based, in which participants are required to be physically active to play (21). Although originally designed for entertainment, exergames that combine digital gaming and physical activity are increasingly used for health promotion including physical (22–26), cognitive (23, 24, 27), and emotional well-being (26, 28). Exergaming is a social activity that provides opportunities for players to interact with each other, which may foster connectedness and reduce loneliness. Such social benefits of exergame play are drawing increasing interest from the research community (29–31), and exergames show promise for enhancing social well-being among older adults (32). Not only do exergames show promise in reducing loneliness, increasing social connection, and fostering positive attitudes toward others (26, 32, 33), but exergames may provide opportunities for social engagement with peers and family members (34). Intergenerational play may be particularly beneficial for older adults, as gaming with a younger partner significantly reduced older adult loneliness compared to passive television viewing with a younger partner (28). The positive social benefits may even extend to observers, as older adult spectators reported enjoying “cheering on” their gaming peers (34).

Exergames are a viable tool to address social isolation and loneliness among older adults, but more research on the exergaming experience is warranted. Before exergames can be prescribed as a way to combat social isolation and loneliness, especially in times when social distancing is required, it is imperative to first establish older adults' thoughts and experiences regarding these games. Specifically, research is needed to understand whether older adults like exergames, which components of exergames they like and dislike, and if there are subgroups of older adults who are more likely to enjoy exergames. If older adults enjoy the exergames they are playing, they are likely to engage more with the games and receive any potential social engagement benefits. If older adults do not enjoy the exergames or certain aspects of the games, they are likely to not play and will not receive such benefits. The current pilot study will examine older adults' perceptions of two commercially available exergames using qualitative and quantitative approaches. First, the current study will describe older adults' preferences for and attitudes toward exergames. Specifically, it will assess whether older adults prefer one game over another, which elements of gameplay they like/dislike, and whether they believe playing with a partner would make exergame play more enjoyable. Second, the study will explore variations in preferences for and attitudes toward exergames by age, gender, personality, and prior technology experience.

## Materials and Methods

The Feasibility and Enjoyment of Exergames (FLEX) study is a pilot study of 20 healthy community-dwelling older adults conducted between July 2019 and February 2020. The purpose of the FLEX study was to explore the feasibility of an exergame system for use by community-dwelling older adults in a future larger intervention trial. Participants were recruited in a small town in Pennsylvania from flyers in local community spaces (e.g., coffee shops, senior centers) and from a recruitment database of older adults residing in the local community who were interested in being contacted for research studies. Eligible participants completed a take-home packet prior to a 75-min lab visit. Participants between the ages of 65 and 85 who were willing and able to do moderate to vigorous physical activity were included in the study. Exclusion criteria included: residing in a nursing home or other institution; being older than 85 or younger than 65 years of age; having no English language proficiency; having participated in an organized exercise program for more than 2 h/week in the past 2 years; using a video game console for more than 2 h/week in the past 2 years; using a walker, cane, and/or wheelchair; having more than two falls in the past 2 months; reporting Parkinson's disease or other motor diseases, uncontrolled asthma, COPD, peripheral neuropathy, diabetes, cardiac disease, or hypertension; having a history of traumatic brain injury; being advised by a medical professional to not do moderate to vigorous physical activity; and having a Memory Impairment Screen-Telephone score of 4 or lower (35). This study was approved by the Pennsylvania State University Institutional Review Board and has been preregistered on Open Science Framework^1^, where detailed study information can be found.

### Exergames

This study utilized two commercially available exercise video games, Just Dance and Kinect Sports Rivals, on the Xbox One with Kinect console. Exergames were played on a sixty-inch 1,080 p LED television. Both exergames are controlled by participant movement by the Kinect console's motion-sensing camera. Just Dance is a dance-based exergame where participants mimic the dance movements of the on-screen character. Participants danced to three songs for 3 min each. Game points are rewarded based on their dance movement accuracy. Kinect Sports Rivals is a sports-based exergame where participants competed in three sporting events for 3 min each: bowling, tennis, and target shooting. The exergames were set up prior to the lab visit so participants did not have to navigate any screens. Participants played each exergame for 10 min, with a 5-min break offered in between gameplay. Game presentation was counterbalanced such that half participants (*n* = 10) played Just Dance first and half played Kinect Sports Rivals first. The research assistant observed gameplay and offered instructions as needed.

### Measures

#### Personality

Personality was assessed during the in-person visit before exergame play using the Big Five Inventory 44-Item (36). The Big Five Inventory is a self-report questionnaire that assesses five personality traits (Openness, Conscientiousness, Extraversion, Agreeableness, and Neuroticism). The current study included Extraversion and Openness to Experience as predictors of exergame experience.

#### Mobile Device Proficiency

As part of the screening process, participants did not have prior experience with console video games. To assess prior technology experience, a measure of mobile device proficiency was included in the current study in the take-home questionnaire. The Mobile Device Proficiency Questionnaire (MDPQ) is an eight-item questionnaire which assesses proficiency in four areas of mobile device usage: Mobile Device Basics, Communication, Data and File Storage, Internet, Calendar, Entertainment, Privacy, and Troubleshooting, and Software Management (37). Proficiency scores ranged from 1 to 5 with the lowest rating having “never tried” the listed action on a mobile device and the highest rating indicating they can “very easily” carry out the action on a mobile device (i.e., “Using a mobile device I can setup a password to lock/unlock the device”). The total MDPQ score is the sum of the averages of the four subscales, with possible scores ranging from 0 to 20.

#### Experiences With Exergame

Participants' experiences with the exergames were measured quantitatively and qualitatively. Participants provided quantitative feedback by responding to the following items on a scale of 1 to 5, where higher score represent more favorable opinions: (1) enjoyment of the exergames (“Did you enjoy the exergame?”), where 1 = disliked and 5 = greatly enjoyed; (2) motivation during game play (“How motivated were you to do well on the exergame?”) where 1 = no motivation and 5 = highly motivated; and (3) likelihood of playing the exergame in the future (“How likely are you to play an exergame like the one you just played in the future?”) where 1 = highly unlikely and 5 = highly likely. Participants also reported how likely they were on a scale from 0 (very unlikely) to 3 (very likely) to say, “I feel like I have the money to play a game like this in my home.” Participants provided qualitative feedback by writing their responses to the following items: “What was the most enjoyable part of the exergame?,” “What was the least enjoyable part of the exergame?,” and “Would playing with a partner make exergaming more enjoyable?.” For the last item, all participants wrote some version of “yes,” “no,” or “maybe,” so this item was converted into a quantitative item where a score of 1 indicates yes/maybe and a score of 0 indicates no.

#### Current Physical Activity

To characterize participants' current levels of physical activity, we used the Rapid Assessment of Physical Activity (RAPA) assessed at the baseline visit (38). The RAPA is a nine-item questionnaire that measures one's usual aerobic and strength/resistance physical activity engagement. Higher scores indicate a greater level of physical activity engagement.

#### Mild Cognitive Impairment

The Montreal Cognitive Assessment (MoCA) was administered during the study visit to assess potential mild cognitive impairment (MCI) or dementia (39). The MoCA is a rapid cognitive screening test designed for MCI or dementia detection. It specifically assesses attention and concentration, executive functions, memory, language, visuoconstructional skills, conceptual thinking, calculations, and orientation. Higher scores are thought to reflect normal cognitive function.

### Analytic Plan

To accomplish Aim 1 (describe older adults' preferences for and attitudes toward exergames), means and standard deviations were calculated for each quantitative measure of exergame experience. Qualitative feedback on exergames is presented in-text to identify elements of gameplay that older adults liked and disliked. To accomplish Aim 2 (explore variations in preferences for and attitudes toward exergames), scores on quantitative measures of exergame experience were compared by gender, age, technology experience, extraversion, and openness to experience. To assess gender differences in continuous outcomes, *t*-tests were conducted. To assess the association between continuous outcomes and age, technology experience, extraversion, and openness to experience, Pearson correlations were conducted. To assess differences in whether playing with a partner would make exergaming more enjoyable by age, technology experience, extraversion, and openness to experience, point-biserial correlations were conducted. Chi square analysis was used to assess gender differences in this item. Significance values were set at *p* < 0.05 and all analyses were conducted in SPSS 26.

## Results

### Participants

Thirty-eight participants were screened, and 20 met inclusion criteria and were enrolled in the study. Table 1 highlights demographic information for the study sample. Most participants were female (80%), White (95%), had a college degree (85%), and were smartphone users (85%). The average age of the sample was 73.03 years old (5.95) and the average MoCA score was 26.05 (2.62). Most participants reported participating in aerobic physical activity (65%), and 45% reported being physically active in regards to strength and flexibility as assessed by the RAPA.

**Table 1:** Demographics of study sample (*N* = 20).

	***M* (*SD*) or %**	**Range**
Age	73.30 (5.95)	65–84
MoCA	26.05 (2.62)	20–30
Gender (Women)	80%	
Race (White)	95%	
College degree or higher	85%	
Smartphone owner	85%	
Physically active (aerobic)	65%	
Physically active (strength and flexibility)	45%	

### Quantitative Feedback

**Figure 1:**
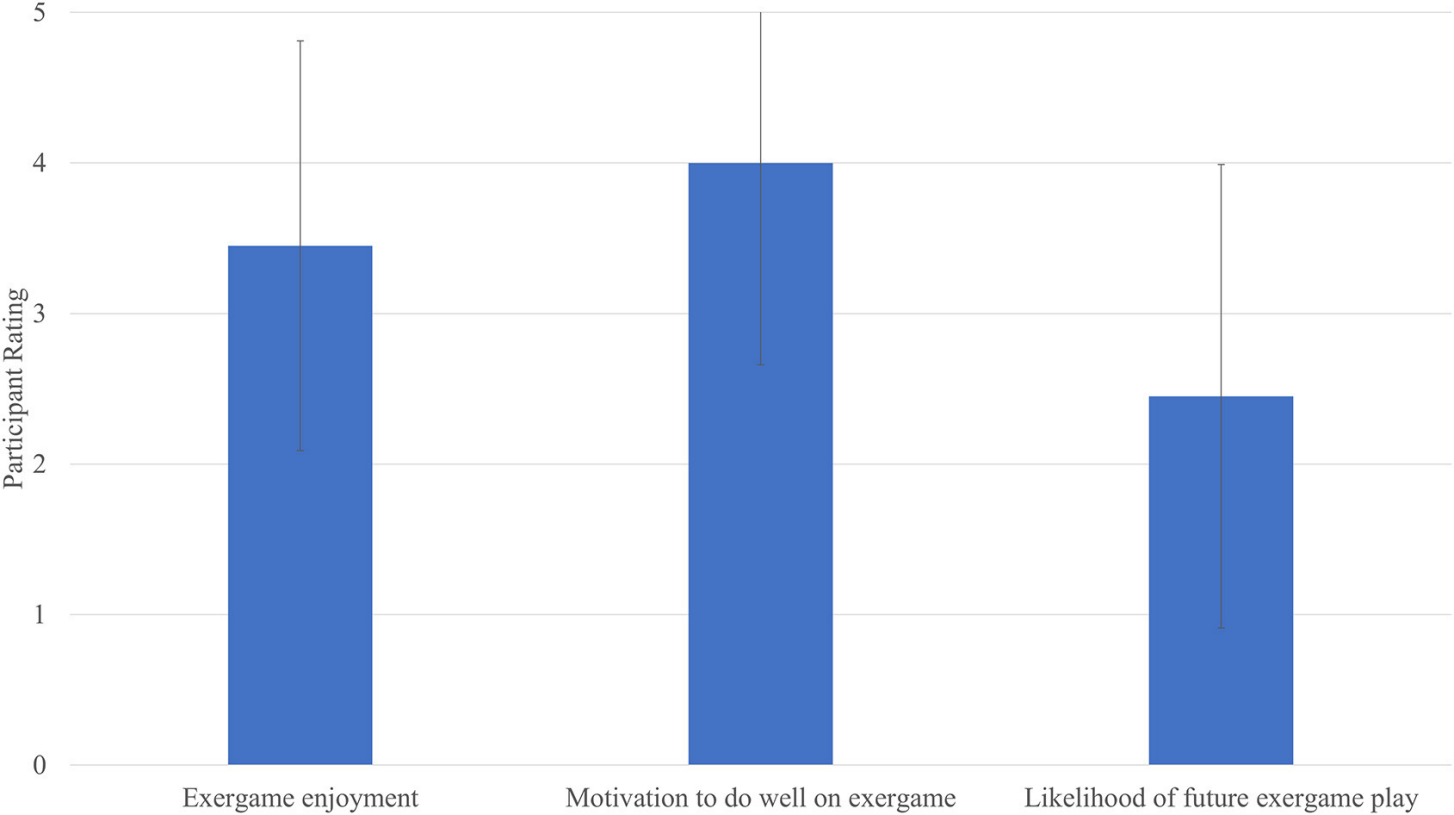
Participants' quantitative feedback on exergames (*N* = 20). Higher scores indicate more favorable responses. Bars indicate mean rating and lines indicate standard deviations for each item.

Means and standard deviations for quantitative items are presented in Figure 1. Participants reported an average of 3.45 out of 5 (*SD* = 1.36) for exergame enjoyment, though responses ranged from disliked (1) to greatly enjoyed (5). They were highly motivated to do well on the games (*M* = 4.0, *SD* = 1.34) but reported an average of 2.45 out of 5 for likelihood of playing these games in the future. Eight participants responded with a score of 1 indicating that they were “highly unlikely” to play exergames like the ones they just played in the future. Participants were also asked if the cost of an Xbox and the games would impact their likelihood of buying the games; equal numbers of participants said yes (45%) and no (45%) and two participants said “probably.” Most participants (80%) were somewhat likely or very likely to say that they have the money to play similar exergames in their home. The majority of participants (65%) said that they would not be likely to buy this game for themselves. Thirteen out of 20 participants said that playing with a partner would make exergaming more enjoyable.

Next, the current study examined whether participant characteristics were associated with enjoyment, motivation, likelihood of future play, and whether playing with a partner would make exergaming more enjoyable. There were no significant gender differences in enjoyment [*t*_(18)_ = 0.49], motivation [*t*_(18)_ = 0.41], likelihood of future play [*t*_(18)_ = 0.43], or indicating that playing with a partner would make the games more enjoyable, $\chi_{\left(1,\text{N}=20\right)}^{2}$ = 2.69, *p*'s > 0.05. Correlation results are presented in Table 2. Correlations between exergame experience and age were large: Older adults reported significantly lower enjoyment (*r* = −0.53), motivation (*r* = −0.68), and likelihood of future play (*r* = −0.55). There was also a large correlation between greater prior technology experience and saying that playing with a partner would not make the exergame more enjoyable (40). However, prior technology experience was not significantly correlated with enjoyment, motivation, or future play. Out of the two personality measures, only extraversion was associated with a measure of exergame experience; older adults scoring higher on extraversion reported greater enjoyment of the exergames (*r* = 0.56). Openness to experience was not significantly associated with any measure of exergame experience.

**Table 2:** Correlations between demographic variables and exergame experience.

	**Enjoyment**	**Motivation**	**Future play**	**Partner play^a^**
Age	−0.53^*^	−0.68^*^	−0.55^*^	0.34
Technology experience	0.36	0.37	0.34	−0.54^*^
Extraversion	0.56^*^	0.39	0.35	−0.22
Openness to experience	0.28	−0.03	−0.27	−0.22

a *Point-biserial correlations. Levene's Test p's > 0.05*.

* *p < 0.05*.

### Qualitative Feedback

Participants were asked to report the most enjoyable and least enjoyable parts of the exergame experience. Seven participants said Kinect Sports Rivals was the most enjoyable, while five said it was the least enjoyable. Five participants indicated that Just Dance was the most enjoyable part, while six said it was the least enjoyable part. Participants also wrote responses not specific to either game, such as saying the most enjoyable part was “winning” and that the games were “fun to play.” Responses for “least enjoyable” included “not being very good at them,” “standing in one spot,” and “all parts.” One participant wrote, “I was totally turned off by the graphics, noise, musical distractions in the three games.”

Finally, we asked participants to provide any additional thoughts they had on the exergames and what factors may influence these thoughts. A little over half of the participants had no comments to add. Responses to this item include, “Not aerobic or strengthen enough; not enough exertion” though one person wanted “slower movements, repetition, clearer purpose of doing the games.” One participant wrote “Not inclined to have yet one more electronic gadget in my house that I would probably lose interest in.” Another participant wrote “If the video graphics and music were geared more to Boomers it would be a possibility.” This person did not like the graphics and sound in the game, indicating that it was “too much.” Three participants requested to stop exergame play prior to the 10-min play period; all three participants stopped play during Just Dance.

## Discussion

This pilot study explored older adults' perceptions of two commercially available exergames. Overall, participants' enjoyment of the games was mostly neutral despite being motivated to perform well, and they generally reported they were unlikely to play these games in the future. Participants were about equally split on some opinions about the exergames. For example, some participants said Kinect Sports Rivals was the most enjoyable, while other participants said Just Dance was the most enjoyable. Some participants thought the games were not difficult enough, while others thought the games should be slower. The current study also found that participants' characteristics were associated with their experiences. Older age was moderately associated with lower ratings for enjoyment, motivation, and likelihood of future play. Extraversion was strongly associated with greater enjoyment, and prior technology experience was associated with not saying that playing with a partner would make the games more enjoyable. Gender was not associated with any measure of exergame experience. The results of this small pilot study suggest that exergames may be a possible tool that can be used in older adult populations. If so, such games may help address social isolation and loneliness among older adults particularly during times of social distancing and indicate possible avenues for future research on this important issue.

The current study's findings are similar to other studies of older adults' perceptions of commercially available video games (41, 42). The current study found similar neutral amounts of enjoyment and mixed feedback regarding game difficulty for these commercially available games. The Xbox with Kinect games played in this study were not specifically designed for older adults and this was likely reflected in their feedback. In other studies using exergames specifically developed for older adults, older adults had higher ratings for overall enjoyment and motivation to do well (43, 44). While commercially available games are a cost-effective and quick way to administer interventions, games not designed specifically for older adults may not be as well-received as games designed with older adults in mind.

An important finding in the current study was that game preferences varied. Some participants thought the games were too difficult, while others thought the games were too easy. Because of the nature of this pilot study, findings regarding age, gender, and personality differences in game experience cannot be generalized to the larger population. However, the results have important implications for future research on exergames in this population. Larger, more representative studies should evaluate whether personal characteristics such as age, gender, and personality play a role in people's experiences with exergames.

Exergames could also be effective for groups of older adults who are particularly at-risk for social isolation and loneliness based on characteristics not assessed in the current study. For example, older adults who live alone, do not regularly engage with groups in their community, have physical health issues, and lack connection with close friends or family members are at the highest risk of not being socially connected (45).

Importantly, the findings of the current study suggest that older adults may be receptive to playing exergames with a partner. Over half of the sample indicated that playing with a partner would make exergames more enjoyable. Prior work has found that older adults in nursing home and assisted living settings enjoy the multiplayer components of commercially-available exergames (26, 34, 46). One promising avenue to increasing social connectedness without requiring physical proximity is the use of online multiplayer features of exergames. The exergames played in the current study allow players to interact with other people online. When older adults cannot gather in-person to play games, online multiplayer play would allow them to play games with friends while remaining socially distanced. This option may also benefit areas with limited activity or transportation access such as rural communities. Future work should examine whether the social benefits of multiplayer exergames extend to online formats.

Despite its potential health and psychological benefits to older adults, there are concerns about exergame use in this population. The American College of Sports Medicine encourages older adults to engage in physical activity that incorporates flexibility and balance with slow movements (47). Such activities occur in exergames like Wii Bowling, where users can dictate the speed of movement without negatively impacting their performance. Other activities such as Just Dance, however, require the user to maintain a particular pace in order to perform well on the activity. Games de-emphasizing speed, or those which increase speed demands slowly, may be more appropriate, especially as users become familiar with the gaming system. A related concern of exergames is their use among those with physical limitations. Older adults with physical limitations frequently report social isolation (48), and early evidence suggests exergames may promote social health among those with physical disabilities (26). Despite promising benefits, exergame safety among this population should be considered. Adverse health events due to an exergaming intervention are infrequent, but injury is possible (49). Furthermore, exergames do not always accurately track and register user movement, which can make gameplay frustrating. If the game incorporates a speed element, this may exacerbate a physically-limited user's frustration and decrease self-efficacy, motivation, and enjoyment of the gaming system. As exergames are not intentionally designed for older adult gameplay, it is important to understand adverse events and gameplay experiences among this group, as health care providers and exergame developers should take these into consideration when developing and implementing exergame programs.

This study provides important quantitative and qualitative data about older adults' initial experiences with and perceptions of selected commercially available games. However, there are some limitations worth noting. First, the FLEX study is a small pilot study designed to inform larger future observational and intervention studies. The sample was also relatively homogenous, and most participants identified as white and as women. The percent of white participants was similar to that of the county where participants were recruited (87% white), though the study sample was over-represented by women (80% in study sample vs. 54% of people 65 and older in the population) (50). The lack of gender and racial diversity in the sample limits the ability of study findings to be generalizable to the older adult population at large. Additionally, we could not examine differences in exergame preferences and experiences by educational attainment because all but three participants completed a college degree or higher, compared to 45% of the county's population of adults.

There were also only two exergames examined in the study, while there are many commercially available exergames that may have benefits. This pilot study also was limited in the time participants could learn and play the exergames; it is possible that participants' experiences with the exergames would shift over more gameplay sessions. Finally, the current study was not a training study so social isolation and loneliness were not assessed before and after exergame play. Therefore, no conclusions can be made about the efficacy of such games for reducing social isolation and loneliness. Future research should examine a wider selection of games in diverse and larger samples. Relatedly, more precise examination of specific elements that improve game satisfaction for diverse older adult samples are needed. Future research should examine the development of games that are attractive to a range of older adults and also include evidence-based components to maintain health, social engagement, and well-being. Though the exergames were already set up prior to participants' lab visit, there was limited time during the lab visit to provide instructions and allow participants to practice exergames. This may have contributed to some participants' negative experiences with the exergames reported in quantitative and qualitative feedback.

Exergames have the potential to improve health and decrease social isolation and loneliness in older adults. Games that can be played online may allow for social distancing while providing social connections. The current study lays the foundation for future, larger scale studies on older adults' perceptions of exergames, including comparing commercially available exergames to games designed specifically for older adults, offering different levels of gameplay difficulty, and exploring how exergame play by older adults can be supported remotely. Beyond the COVID 19 pandemic, some older adults may continue to be physically isolated from others for a number of reasons, such as difficulties with transportation. Exergames have the potential to address social isolation and loneliness by providing the opportunity for leisure and physical activity while being socially connected to others if designed in a way that promotes engagement.

## Data Availability Statement

The raw data supporting the conclusions of this article will be made available by the authors, without undue reservation.

## Ethics Statement

The studies involving human participants were reviewed and approved by Penn State University Institutional Review Board. The patients/participants provided their written informed consent to participate in this study.

## Author Contributions

SF, BS, and LR contributed to the study design and execution. SF and CD carried out all primary data collection. SF, BS, LR, CP, CD, JT, and AS contributed to writing the manuscript and provided critical feedback. All authors reviewed the results and approved the final version of the manuscript.

## Funding

This work was supported by the Pennsylvania State University College of Health and Human Development Small Projects Grant and the Study of Healthy Aging and Applied Research Programs (SHAARP) lab.

## Conflict of Interest

The authors declare that the research was conducted in the absence of any commercial or financial relationships that could be construed as a potential conflict of interest.

## Publisher's Note

All claims expressed in this article are solely those of the authors and do not necessarily represent those of their affiliated organizations, or those of the publisher, the editors and the reviewers. Any product that may be evaluated in this article, or claim that may be made by its manufacturer, is not guaranteed or endorsed by the publisher.

## Footnotes

^1^ https://osf.io/y8as7/registrations
